# Black eyes following stem cell transplantation

**DOI:** 10.1002/jha2.584

**Published:** 2022-11-14

**Authors:** Osamu Imataki, Tomoya Ishida, Haruyuki Fujita, Makiko Uemura

**Affiliations:** ^1^ Division of Hematology Department of Internal Medicine Faculty of Medicine Kagawa University Kagawa Japan

**Keywords:** acute lymphoblastic leukemia, black eyes, stem cell transplantation, thrombocytopenia

1

We treated a 32‐year‐old Japanese female patient with Philadelphia chromosome‐negative acute lymphoblastic leukemia, whose disease relapsed during maintenance therapy after achieving the first remission. The patient underwent stem cell transplantation (SCT) from a human leukocyte antigen‐full matched unrelated donor with a preparation consisting of middle‐dose etoposide (30 mg/kg), cyclophosphamide (120 mg/kg), and total body irradiation (12 Gy). Then, the patient obtained neutrophil engraftment on day 16 of treatment; however, thrombocyte and erythrocyte counts remained low, warranting platelet and erythrocyte transfusion.

Between days 5 and 12 following SCT treatment, the patient experienced severe emesis and strain during retching. Meanwhile, the patient's platelet count dropped to < 20 × 10^3^/μl, for which she received a daily dose of 10 U of platelet transfusion. Until day 17, the patient's skin around the bilateral orbital formed patchy purpura (Figure 1) due to repeated retching from emesis, which was caused by mucositis after SCT. It presented in the form of raccoon or panda eyes.

**FIGURE 1:**
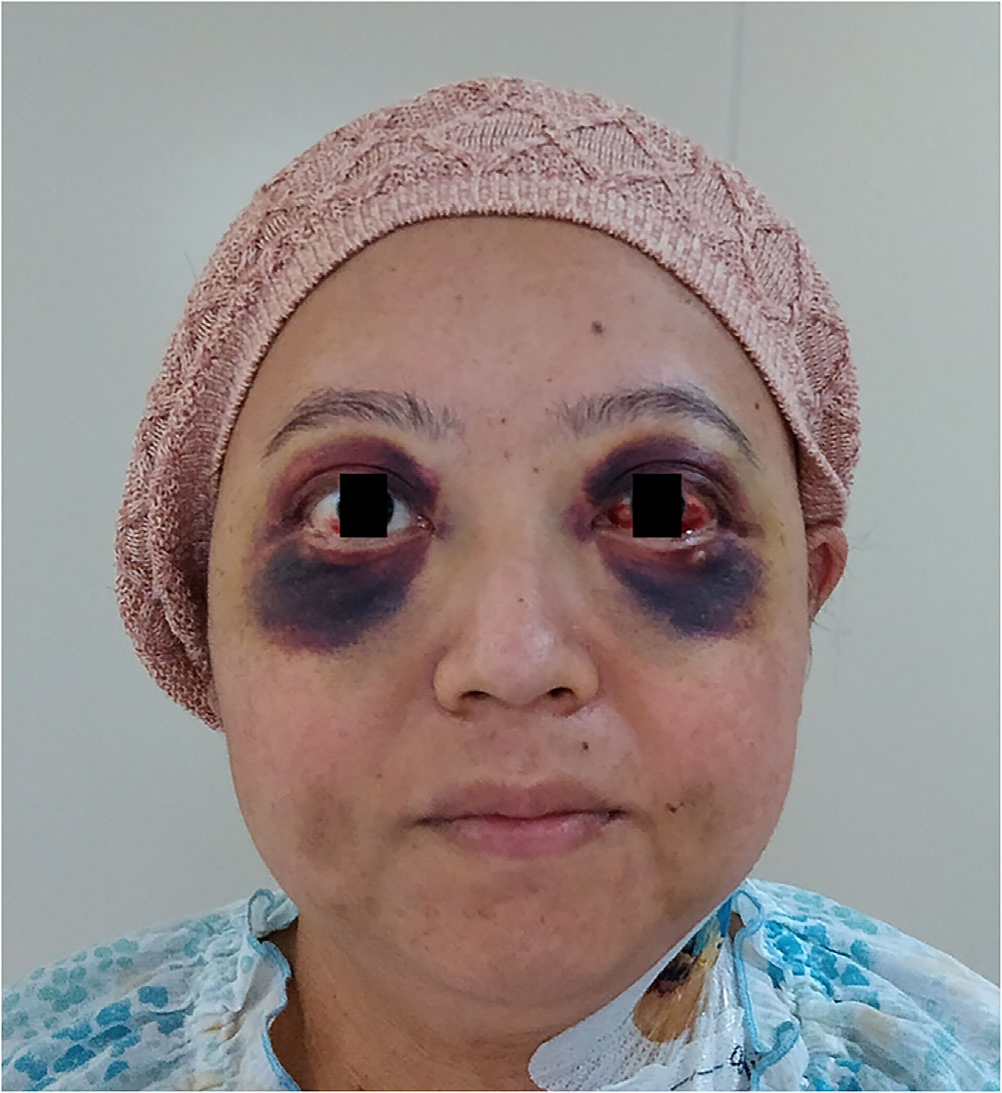
The patient's purpura around the bilateral orbita

“Black eye” is a common feature of unilateral periorbital bruising. The leading cause of black eye is an eye injury. Black eye is also caused by severe vomiting and coughing. Repeated straining during retching may increase intrathoracic pressure. Transient increases in intrathoracic pressure lead to venous congestion of the facial vein draining from the periorbital veins. Moreover, in this case, dyscoagulopathy (thrombocytopenia after SCT) enhanced the bleeding tendency. This is a proposed physiological mechanism [1]. The patient's kawaii (“cute” in Japanese) sign resolved spontaneously before day 30.

## CONFLICT OF INTEREST

The authors declare that they have no conflict of interest.

## FUNDING INFORMATION

This work was supported by internal funding, JSPS KAKENHI Grant Numbers JP19K17927. We don't have any other financial relationships to disclose.
